# Genotoxic Effects of Silver Nanoparticles on Mice in Vivo 

**Published:** 2009-10

**Authors:** C.G. Ordzhonikidze, L.K. Ramaiyya, E.M. Egorova, A.V. Rubanovich

**Affiliations:** 1 Vavilov Institute of General Genetics, Russian Academy of Sciences;; 2 Institute of General Pathology and Pathophysiology, Russian Academy of Medical Sciences;; 3 Science-Technology Company "Nanomet"

## Abstract

The toxic and genotoxic effects of silver nanoparticles were studied on injected mice (BALB/c line) in vivo. A water solution of silver nanoparticles (SNP) with particle sizes of 9±6 nm was obtained by means of the original method of biochemical synthesis. The effect of the SNP solution was compared to those of AOT (anionic surfactant used as SNP stabilizer) and silver nitrate (i.e. Ag+ ions) introduced as water solutions. In studies of the toxic effects, the death of mice was registered 12-24 hours after injection only at two maximum dozes of SNP (equivalent to 0.54 and 0.36 gAg/l). It is shown that the toxic effect decreases in the sequence SNP>AOT>>AgNO3. The LE50/30 values for SNP and AOT are equal to 0.30±0.07 gAg/l and 13.3±2.1 gAg/l, respectively. Genotoxic effects were assessed by the abnormal sperm heads test and neutral Comet assay. The frequencies of abnormal sperm heads (ASHs) did not differ after treatment by SNP and AOT, but both were significantly higher than those found with AgNO3 and in control mice. Comet assay showed an increase of the DNA percentage in the comet tail in spleen cells after the injection of SNP and AOT in concentrations of ≤ LE50/30. Tail DNA % was 32.8±1.3 and 26.3±1.7%, respectively, vs 16.2±0.7% for the untreated control. To sum up, these tests showed that the genotoxic effects of the SNP solution are associated with the presence of AOT rather than SNP.

## Introduction

The use of nanotechnological products in human activities has been steadily increasing in recent years. Because of this, it is of vital importance to study the biological effect of various nanoparticles and nanocomposite materials, and especially their effects on animal and human organisms. The main issue is to elicit the toxicity of nanoparticles for humans and thus, the potential risk in the use of nanoparticle- and particle-based products.

Of utmost interest are metal nanoparticles and their biological effect, since these particles are most often used in novel products in various fields in manufacturing and medicine. During the last decade, abundant data has been obtained on both the positive (therapeutic effect) and negative (increase in the appearance of various diseases) effects of metal nanoparticles on living organisms [1, 2]. Silver nanoparticles are one of the most popular objects of research, since they have been actively used in the manufacturing of various consumer goods, such as dietary supplements, clothes, household appliances, toys, etc. Silver particles were mainly studied in bacteria, so as to elicit the particle’s antimicrobial activity [3-5] or using in vitro cell cultures (for example [6]). There are also some data on the effect of silver nanoparticles on human fibroblasts [7]. Over all, data on the effect of silver and other metal nanoparticles are sparse. Up to now, there have been practically no data on the biological and genetic effect of silver nanoparticles introduced into a mamMalian organism. 

## Experimental procedures 

This work studied the toxic and genetic effects of silver nanoparticles (SNPs) on mice in vivo. We used water dispersions of SNPs obtained by biochemical synthesis [8]. The particle size was 9 ± 6 nm. It was previously shown that water dispersions showed a pronounced antibacterial and antiviral effect [9], as well as a strong antibiotic effect on the slime mold Physarum polycephalum [10].

The initial concentration of dispersed SNPs (further named SNP preparation) was 5 · 10-3 g·ion/l as calculated for Ag+. The effect of the SNP preparation was compared with that of the dispersant---an anionic surfactant (AOT), which acts as a stabilizer of the SNP preparation---and also with the effect of Ag+ ions in equivalent concentrations. Aqueous solutions of AOT (initial concentration - 15 mM) and silver nitrate (initial concentration - 5 mM) were used.

The tests were conducted on male and female laboratory mice of the BALB/c line. The mice were 3-4 months of age and weighed 30-35 g. In order to determine the survival rate and lethal dose (the dose that causes the death of 50% of the subjects), the animals were divided into 4 groups of 16 mice each. The mice of the first group received a single intraperitoneal injection of the SNP solution (in distilled water, 0.2 ml). The concentration of SNP was varied by dilution of the initial preparation 0; 1.5; 2; 3; 5; 7; 10; and 100-fold. Thus, the SNP concentrations in the solutions were 5; 3.3; 2.5; 1.6; 1.0; 0.7; 0.5; and 0.05 · (10-3 g·ion /l).

The second group was injected with AOT solutions in accordance with the dosages in Group 1. The equivalent concentrations of AOT for the above-mentioned SNP solutions are 15; 10; 7.5; 5.0; 3.0; 2.5; 1.5; and 0.15 mM. 

Mice from Group 3 were injected with an aqueous solution of AgNO3 at 5; 0.5; and 0.05 mM concentrations, which is equivalent to the 0; 10; and 100-fold SNP dilutions.

Group 4 was a control group and was injected with 0.2 ml of distilled water. 

The lethal effect of the injected solutions was determined according to the standard protocol. All the injected mice were kept in an animal facility for 30 days, and animal death was monitored daily. Observation of the physical condition of the animals showed that during the first hours after injection, mice from Group 1 with the highest dosages (5 and 3.3 · 10-3 g·ion/l) of SNPs exhibited a decreased motor activity and convulsions, followed by paralysis of the hind limbs. The animals died 12-24 hours after injection of the preparation. The other mice from this group with a lower dosage of the drug showed less prominent motor depression and toxic syndrome during the first few hours after injection, and the overall condition of these mice did not differ noticeably from the control animals. Mice in this group also died despite lowered concentrations: however, the survival time of these animals was greater (9-10 days) than that of the animals that received higher doses (1-3 days).

In Group 2, mice died only at high doses of AOT (15; 10; and 7.5 mM). Lower concentrations of AOT did not cause animal death. Groups 3 and 4 did not exhibit any animal death during the 30 days of observation. 

**Fig. 1.**
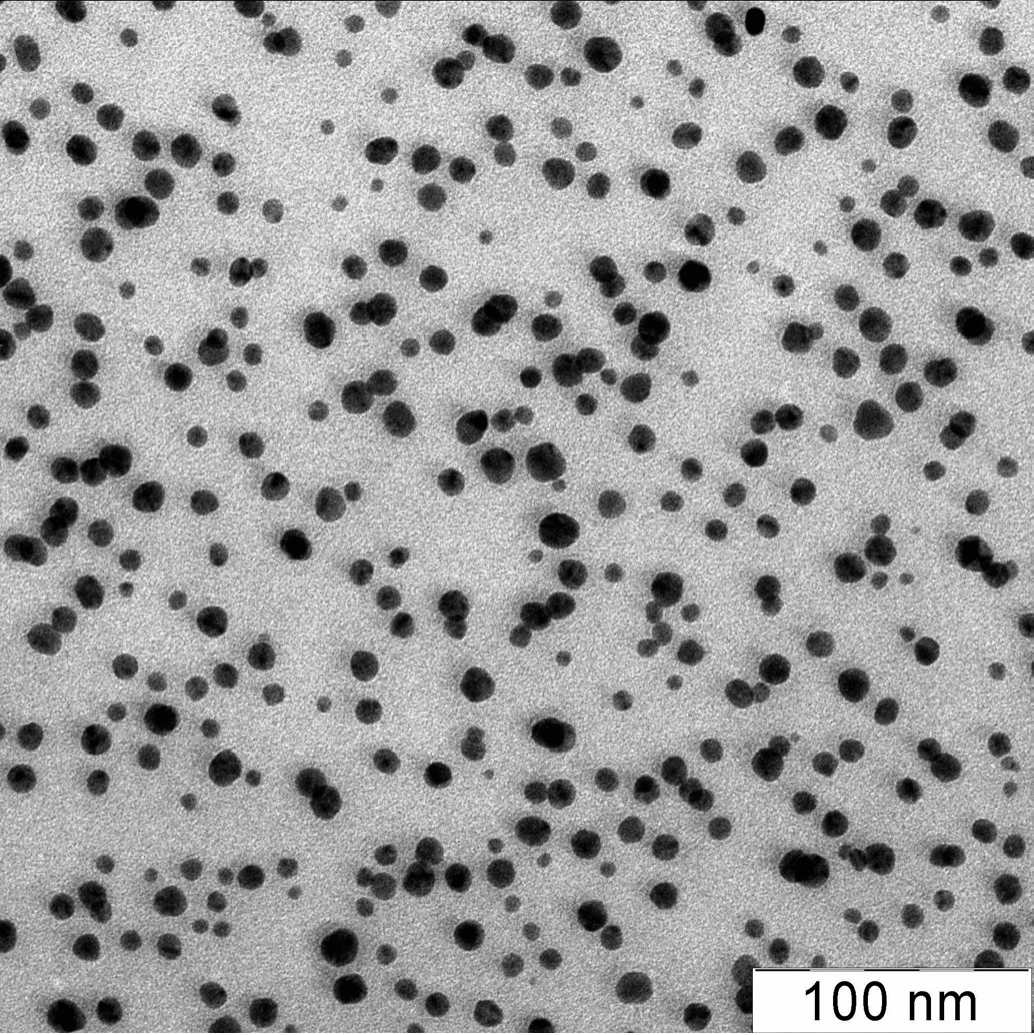
Electronic microphotograph of silver nanoparticles

The relationship between mouse death and the concentration of the injected solution is presented in Fig. 1.

The data presented in Fig. 1 show that a mouse’s survival rate decreased monotonously as the concentration of the SNP solution was increased, with the exception of the lowest concentration (0.05 · 10-3 g·ion/l - 100-fold dilution). Regressive analysis yielded LD50/30 figures for the preparations studied. LD50/30 for the SNP preparation was (2.74 ± 0.67) ≤ 10-3 g≤ion/l at an AOT concentration of 8.3 mM (1.8-fold dilution). Extrapolation of these data shows that LD50/30 for AOT without silver nanoparticles is 29.9 ± 4.8 mM. Thus, we can assume that the toxic effect of the dispersed silver nanoparticles is 3.6-fold stronger than that of the dispersant. The absence of animal death in the group that received silver nitrate injections shows that, of the three studied reagents, Ag+ ions have the lowest toxic effect. Thus, the toxic effect decreases in the following sequence: SNP> AOT >> AgNO3 .

In order to study the effect of the SNP preparation on mamMalian reproductive cells, we monitored the number of emerging sperm with anomalous head morphology (AHS) 21 days after injection of the preparation. This method allows to detect the deleterious effects of a preparation on reproductive cells in the early premeiotic stage of gametogenesis, namely the pachytene of the first meiotic division. It is assumed that AHS emergence is caused either by gross chromosomal aberrations, such as translocations, or by point mutations and small deletions or, alternatively, by somatic damage. It was shown earlier that certain physical (ionizing radiation, microwaves) and chemical factors (cyclophosphamide, cadmium chloride, zinc chloride, etc.) increased the AHS index and also caused a decrease in testicular mass, an increase in pre-implantation pregnancy loss and a decrease of effective copulations caused by damage to premeiotic male reproductive cells in mice. These data point not only to a mutagenic, but also to a cytolytic and/or cytotoxic effect of these factors [11]. The method for determining the AHS index is fairly simple; it does not require a large number of animals and can be used as an estimate of the mutagenic effect of various drugs [12].

**Fig. 2.**
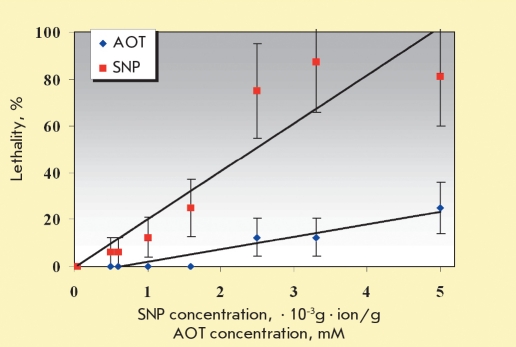
Death rate dependence on the concentrations of SNP and AOT for injected mice

Figure 2A shows AHS index data obtained after injection of AOT and SNP solutions into male mice. The AOT and SNP concentrations were 5.0 mM and 1.6 · 10-3 g·ion/l, respectively (the initial samples were diluted 3-fold). The data presented in Fig. 2A indicate that the deleterious effect of SNPs and AOT was greater (approximately 1.5 fold) than that of the control injections. Notably, there was no marked difference between the effects of AOT and SNPs.

The same animals were also tested for primary DNA damage using neutral gel-electrophoresis of individual cells (DNA-comets). Damaged DNA (DNA with single and double strand breaks) is a sign of oxidative stress and cell death. The DNA-comet method can provide some information about induced mutagenesis by visualizing the relative amount of damaged DNA. This electrophoretic method is based on the different mobility of the whole DNA and its possible degradation fragments obtained from lysed cells trapped in an agarose gel.

This test was performed according to the standard protocol [13]. We chose the spleen as a target organ because of its specific function in the circulatory system (lymphocytes, monocytes, and macrophages that are accumulated in the white and red pulp can easily be damaged by nanoparticles). 

Using the DNA-comet method, we measured the level of DNA damage after injecting the SNP preparation and the AOT solution (concentrations 1.6 · 10-3 g·ion/l and 5 mM, respectively). We did not detect any increase of the DNA percentage in the comet tails up to 48 hours after the injections. Figure 2B shows the data averaged for 14 mice and 7 time points (3, 5, 7, 9, 12, 24, and 48 hours). The figure shows that the AOT dispersant had a damaging effect on DNA (the portion of DNA that exited the cell into the "comet tail," was 32.8 ± 1.5 %), which is greater than the respective value for SNPs (26.3 ± 1.7 %). Both of these values exceeded the control value (16.2 ± 0.7 %).

It was previously shown for the mold Physarum polycephalum [10] and E.coli bacterium [9] that an aqueous dispersion of silver nanoparticles had a more pronounced toxic effect than equivalent concentrations of silver ions or solutions of the surfactant, which was a component of the SNP preparation.

## Conclusion

This work shows for the first time that dispersed silver nanoparticles obtained via biochemical synthesis have a lethal effect on mamMalian organisms when injected in vivo. The lethal effect of the dispersed nanoparticles is approximately 4 times greater than that of the AOT dispersant alone (See Fig. 1), while injection of equivalent doses of silver ions is followed by 100% survival of the tested animals. Using a calculation taking into account the mouse’s body weight, we estimate that the dose of silver nanoparticles that causes 50% lethality is 1.9 ≤ 10-6 mg per gram of body mass.

**Fig. 3.**
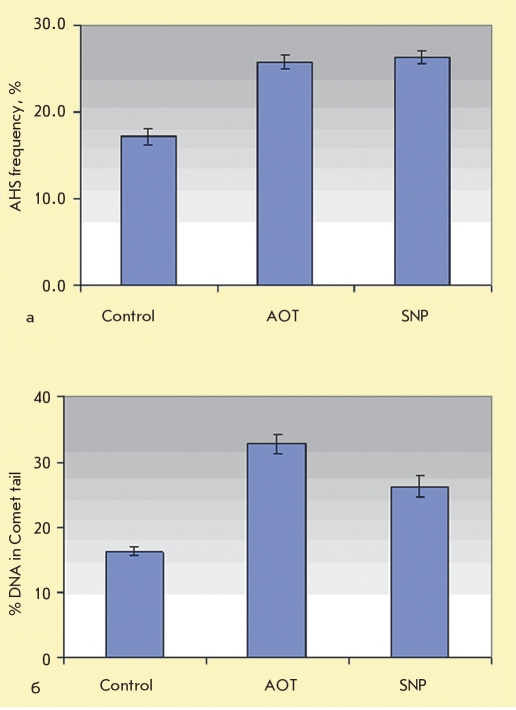
AHS frequency (a) and % of DNA in Comet tail (b) after injection of SNP and AOT solutions (0.17g/l and 2.2 g/l, respectively; dilution in 3)

## Acknowledgements

This work was supported by funding provided by "Nanomet" in accordance with the RAmp;D (contract № 8418-16/09). The authors thank corresponding RAS member prof. N.K. Yankovskiy (IOGEN RAS) for thorough discussion of our results and V.S. Lysenkova, senior technician of the ecological genetics laboratory of IOGEN RAS for technical assistance in this project.
